# Chemodiversity of Dissolved Soil Organic Matter from Amazon Rainforest as Influenced by Deforestation

**DOI:** 10.3390/metabo14030144

**Published:** 2024-02-28

**Authors:** Tancredo Souza, Damiana Justino Araujo, Carlos Alberto Lins Cassimiro, Diego Silva Batista

**Affiliations:** 1 Postgraduate Program in Agroecology, Department of Agriculture, Federal University of Paraiba, Bananeiras 58220-000, Brazil; tancredo_agro@hotmail.com (T.S.); damianaaraujo18@gmail.com (D.J.A.); cassimiro.carlos@insa.gov.br (C.A.L.C.); 2 Centre for Functional Ecology, Department of Life Sciences, University of Coimbra, 3000-456 Coimbra, Portugal

**Keywords:** aromatic compounds, Brazil’s Legal Amazon, deforestation, tropical soils

## Abstract

Many biogeochemical processes are modulated by dissolved organic matter (DOM), but the drivers influencing the chemodiversity of DOM compounds in Amazonian soils are poorly understood. It has also been theorized whether deforestation controls the decline of DOM. In this study, we collected soil samples from thirty sites across different regions of Brazil’s Legal Amazon, and we investigated the trade-offs among soil physical–chemical properties and DOM chemodiversity. We employed optical spectroscopy, Fourier transform ion cyclotron resonance, and multivariate analysis. Our results indicated that, despite variations in land use and soil physical–chemical properties, factors such as the deforested site, geometric mean diameter, weighted average diameter, and soil organic carbon were the main influencers of DOM chemodiversity variation. These findings highlight the importance of considering DOM chemodiversity as closely related to land use and its potential use in developing deforestation models for predicting soil quality decline in Brazil’s Legal Amazon.

## 1. Introduction

In tropical soils, the complex continuum of soluble soil organic matter compounds, also described as dissolved organic matter (DOM), plays a significant role in promoting microbial activity, nutrient cycling, soil fertility, and soil quality [1]. DOM exhibits a complex composition with a high diversity of organic compounds that may be influenced by a wide range of environmental and edaphic factors [2]. However, the influence of deforestation on the chemodiversity of soil DOM in the Amazon basin is poorly understood. 

Some studies have reported that DOM comprises a small proportion of soil organic matter [3], but it is highly susceptible to variations in plant diversity, soil conditions, soil management, land use, and microbial processes [4]. Therefore, understanding the effects of deforestation on the chemodiversity of DOM compounds in tropical soil from Brazil’s Legal Amazon is essential for predicting the decline and dynamics of DOM in tropical soils.

In tropical conditions, the formation of soil organic matter is determined by the partial decomposition and transformation of litter and other plant inputs [5]. In this context, soil DOM represents less than 2% of the total soil organic matter [6]. However, it is the most active portion of soil organic matter and readily accessible to soil organisms [7]. Deforestation can affect the properties of DOM, which in turn may alter microbial functions and reduce the physical protection of soil organic matter [8]. A soil ecosystem with a high diversity of active roots can positively affect the molecular composition and oxidative transformation of DOM in soil ecosystems [4,7].

Deforestation can enhance microbial degradation of certain DOM compounds (e.g., carbohydrates, lipids, and proteins/amino sugars) in tropical soils [9]. Additionally, it has been reported that in deforested sites, there is an increase in the relative contents of recalcitrant compounds (e.g., condensed hydrocarbons, lignin, and tannins) [10]. Meanwhile, deforestation can also affect the composition of DOM by altering soil microbial activities responsible for its decomposition [11]. However, the relationship between the chemodiversity of DOM and deforestation in the Amazon basin, as well as the underlying mechanisms that control the diversity of labile and recalcitrant compounds in tropical soils, is still largely unknown.

The aim of this study was to quantify how deforestation (habitat simplification) affects the soil chemical properties, as well as the composition and diversity of DOM compounds. We hypothesized that plant diversity and root activity play key roles in regulating the chemodiversity of soil DOM in the Amazon basin. Soil DOM was extracted from ten sites in Brazil’s Legal Amazon on a broad landscape scale. Additionally, soil physical–chemical properties and microbial activity were evaluated.

## 2. Materials and Methods

Study site and sampling. In this study, soil samples were collected primarily from three land uses (tropical rainforest, pasture, and deforested sites) to investigate how habitat simplification controls the chemodiversity of DOM in tropical soils. The land uses were characterized by (i) a pasture of *Urochloa brizantha* (Hochst. ex A.Rich.) R.Webster; (ii) a primary Amazon rainforest; and (iii) a deforested site in locations with similar soil type and relief that interacts over time (Table 1). These sites covered a wide range in Brazil’s Amazon rainforest with similar soil type, Acrisols [1,4]. 

Experimental design. We analyzed the influence of three land uses on DOM chemodiversity and soil physical–chemical properties and compared the results with those from the primary Amazon rainforest (a reference area). Before sampling, we followed three main criteria: (i) in each land use, we delimited ten permanent plots (100 × 100 m) with homogeneous stands; (ii) within each plot, we sampled 25 random points in gridded areas (10 × 10 m). Note that no significant effect of site was observed on DOM composition in this study; therefore, we present the mean values per plot; and (iii) each sample included both undisturbed and disturbed samples at a soil depth of 0–20 cm. 

Soil properties. We sampled 300 undisturbed and 300 disturbed soil samples for soil physical–chemical characterization and DOM composition. For physical characterization, we determined aggregate properties: weighted average diameter (WAD) and geometric mean diameter (GMD). Soil bulk density was measured by considering the weight of soil per unit volume of a metallic cylinder [12]. Soil texture was determined as described by IITA [13], using particle size analysis of the dispersed soil. A NaCl solution (Merck, Oakville, ON, Canada) was used as a chemical dispersing agent to flocculate clay [14]. For chemical characterization, soil reaction (pH) was measured in a suspension of soil and distilled water (1:2.5 v:v, soil: water suspension) [12]. Soil organic carbon was determined by the rapid dichromate oxidation method [15]. Microbial respiration was determined by the incubation method, while for the microbial C biomass, we used the fumigation–extraction method [12,14].

DOM preparation and optical characterization. Sampling occurred between September and November 2022 and between September and November 2023. Soil samples were transported to the laboratory within 2 days of sampling, where they were stored at 4 °C in the dark. All analyses including optical measurements and FT-ICR-MS (were conducted within days, and no longer than 2 weeks from collection.

First, soil DOM was extracted by shaking 60 g of dry soil in 0.1 L of deionized water at 25 °C for 24 h. The soil suspension was then collected and centrifuged, followed by filtration through 0.45 μm polyether sulfone filters (Fisher Scientific, Ottawa, ON, Canada), but this filtration does not sterilize the filtrate (e.g., bacteria can pass through such filters). The preservation of samples was checked and no evolution of DOM was observed. In supplement, all supernatant samples were sterilized by UV radiation (Prabhat, Mumbai, Maharashtra, India) to avoid any microbial degradation prior to storage. The filtered supernatant samples were stored at 4 °C prior to optical analyses [16]. 

Portions of the supernatant samples (5 mL) were analyzed for total dissolved organic carbon and used for optical measurements with UV-Vis and three-dimensional excitation emission matrix (3D-EEM) fluorescence spectroscopy (Hitachi F-7000 with a 0.01 m quartz cuvette, São Paulo, Brazil) [17,18]. Since the fluorescence spectra are strongly influenced by pH, we acidified the solution with phosphoric acid (Merck, Oakville, ON, Canada) to pH 2.0, which was the pH value needed for the DOM analyses. The range of values of the emission and excitation scanning were 250–500 nm in increments of 2 nm and 230–500 nm in increments of 5 nm, respectively. Emission and excitation slit widths were both 10 nm, and the scan speed was 1200 nm min^−1^. 

The characteristics of colored and fluorescent fractions of soil DOM were assessed by calculating both optical and fluorescence indices. For optical indices, we included the specific ultraviolet absorbance at 254 nm (SUVA_254_) and the spectral slope (S250-600), which are related to the aromaticity of soil DOM and the apparent molecular weight, respectively [19]. Fluorescence-based indices included the fluorescence index (FI), which is related to the microbial contribution to DOM, and the ratio of emission intensity ranging from 380 to 430 and 450 to 500 nm obtained at excitation wavelengths of 310 and 370 nm, respectively [20]. Additionally, the biological index (BI), related to microbial activity to DOM, was calculated as the ratio of the peak area under the emission spectra of 435–480 nm to that under 300–345 nm plus 435–480 nm at an excitation wavelength of 254 nm [21]. 

FT-ICR-MS analysis. The soil DOM samples were analyzed using a solariX XR FT-ICR-MS (Bruker, Billerica, MA, USA) equipped with a 9.4 T refrigerated actively shielded superconducting magnet and an ESI ion source under negative ion mode [22]. Solid-phase extraction (SPE) was performed on all DOM samples using Varian Bond Elute PPL cartridges (1 g per 6 mL). Briefly, the cartridges were rinsed with 6 mL of methanol (Merck, Oakville, ON, Canada) to maintain the same sampling concentration during the FT-ICR-MS analysis. The detection mass range and ion accumulation time were set to m/z 150–1200 Da and 0.7 s, respectively. Peaks were assigned based on the following criteria: (i) signal-to-noise ratio ≥ 6; (ii) elemental combinations of C ≥ 3, H ≥ 1, O ≥ 1, N ≤ 2, S ≤ 2; (iii) O/C ≤ 1.2; and (iv) H/C ≤ 2.2 [23,24]. 

We normalized the molecular intensity by using the sum of all intensities detected in FT-ICR-MS measurements, enabling us to obtain the relative intensity of each detected molecule [25]. Detected compounds were assigned to main groups based on their molecular formulae to construct van Krevelen (VK) diagrams. Compound groups were delineated based on their modified aromaticity index (AI) [26], H/C, and O/C ratios, including combustion-derived condensed aromatics (AI > 0.66), vascular plant-derived polyphenolic compounds (0.66 ≥ AI > 0.5), highly unsaturated and phenolic compounds (AI < 0.5 and H/C < 1.5), and aliphatic compounds (2.2 ≥ H/C ≥ 1.5) [27]. Furthermore, aliphatic compounds were divided into three components: lipids (H/C: 1.5–2.0; O/C: 0–0.3), proteins/amino sugars (H/C: 1.5–2.2; O/C: 0.3–0.67), and carbohydrates (H/C: 1.5–2.2; O/C: 0.67–1.2). To further characterize the molecular properties of DOM, we estimated the double-bond equivalence (DBE), indicating the number of double bonds and rings in a molecule, and nominal oxidation state of carbon (NOSC), which can be related to the biogeochemical reactivity and bioavailability of a molecule [23]. The relative abundance of each compound group was calculated by summing the relative intensities of all compounds in each compound group. Averages of O/C, H/C, number of O, AI, DBE, and NOSC were calculated for each sample based on the relative intensity of each molecule [24].

Statistical analysis. All variables were analyzed using the Kruskal–Wallis test with land use as the factor and plots as random factors. Bonferroni’s test was employed as the post hoc test (*p* < 0.05). We conducted a principal component analysis (PCA) in R using the ‘rda’ function from the ‘vegan’ package [4] to investigate the distribution of molecular properties among all DOM samples and to assess the potential effects of soil physical–chemical properties and land uses on these properties. All statistical analyses were performed in R 3.4.0 [28].

## 3. Results

The results from the Kruskal–Wallis test showed significant differences among land uses for aliphatic compounds (*p* < 0.001), biological index (*p* < 0.001), carbohydrates (*p* < 0.001), condensed aromatic compounds (*p* < 0.01), fluorescence compounds **1**–**3** (*p* < 0.001), fluorescence compound **4** (*p* < 0.01), fluorescence index (*p* < 0.001), H/C ratio (*p* < 0.001), highly unsaturated and phenolic compounds (*p* < 0.01), lipids (*p* < 0.001), the nominal oxidation state of carbon (*p* < 0.001), polyphenolic compounds (*p* < 0.01), protein/amino sugars (*p* < 0.001), and specific ultraviolet absorbance at 254 nm (*p* < 0.01). The highest significant values for aliphatic compounds, biological index, carbohydrates, fluorescence compounds **1**–**3**, fluorescence index, H/C ratio, lipids, the nominal oxidation state of carbon, O/C ratio, proteins/amino sugars, and specific ultraviolet absorbance at 254 nm were found in the primary Amazon rainforest. No significant differences were found among the land uses for the O/C ratio. Finally, the highest significant values for condensed aromatic compounds, fluorescence compound **4**, highly unsaturated and phenolic compounds, and polyphenolic compounds were found in the deforested site (Table 2). 

The soil physicochemical properties varied significantly among the land uses (*p* < 0.001). The highest significant values of geometric mean diameter, weighted average diameter, soil organic carbon (SOC), microbial carbon biomass, and microbial respiration were found in the primary Amazon rainforest. No significant differences were found among land uses in terms of sand, silt, and clay content. However, the deforested site exhibited the highest values of bulk density and soil pH (Table 3).

**Figure 1:**
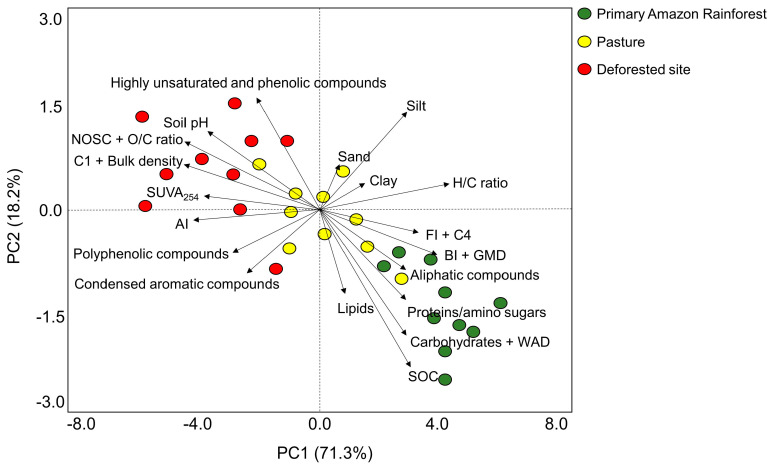
Principal component analysis (PCA) of the distribution of DOM molecular properties among soil samples across the ten studied sites in Brazil’s Legal Amazon. An amount of 71.3% of the variation is explained by PC1, and 18.2% of the variation is explained by PC2. SUVA_254_, the specific ultraviolet absorbance at 254 nm; FI, fluorescence index; BI, biological index; C1 and C4, fluorescence components 1–3 and 4; DBE, double-bond equivalence; AI, aromaticity index; NOSC, nominal oxidation state of carbon; SOC, soil organic carbon; GMD, geometric mean diameter; WAD, weighted average diameter.

Principal component analysis suggested that most DOM properties and soil physical–chemical properties were distributed into three distinct groups (Figure 1). DOM properties exhibited a clear separation between those indicative of the primary Amazon rainforest (e.g., GMD, WAD, SOC, FI, C4, BI, aliphatic compounds, proteins/amino sugars, carbohydrates, and lipids) and those of the deforested site, which were indicative of combustion-derived and plant-derived properties (e.g., bulk density, soil pH, highly unsaturated and phenolic compounds, NOSC, O/C ratio, C1, SUVA_254_, and AI). Correspondingly, DOM samples from pasture were distributed intermediately and distinctly from those from the primary Amazon rainforest and deforested site (Figure 1).

## 4. Discussion

Our results underscored the influence of habitat simplification, as influenced by deforestation, on soil physical–chemical properties and DOM chemodiversity in tropical soils. Essentially, we aimed to understand how the slash-and-burn practice can alter various parameters, including aliphatic compounds, biological index, carbohydrates, condensed aromatic compounds, fluorescence compounds (groups **1**–**3** and **4**), fluorescence index, H/C ratio, highly unsaturated and phenolic compounds, lipids, the nominal oxidation state of carbon, O/C ratio, polyphenolic compounds, proteins/amino sugars, specific ultraviolet absorbance at 254 nm, bulk density, geometric mean diameter, weighted average diameter, sand, silt, clay, soil pH, and SOC. Deforestation had a strong negative influence by decreasing the values of aliphatic compounds, biological index, carbohydrates, fluorescence compounds **1**–**3**, fluorescence index, H/C ratio, lipids, the nominal oxidation state of carbon, O/C ratio, proteins/amino sugars, and specific ultraviolet absorbance at 254 nm. Conversely, this practice promoted an increase in the values of condensed aromatic compounds, fluorescence compound **4**, highly unsaturated and phenolic compounds, and polyphenolic compounds. It is worth noting that both changes in DOM chemodiversity may reduce soil microbial activity and soil nutrient cycling, thereby diminishing soil quality and health [29,30]. Habitat simplification, promoted by pasture and deforestation, strongly influences energy provision and nutrient availability through litter and root diversity compared to the primary Amazon rainforest [4]. Scientific evidence highlights the importance of native/endemic vegetation and labile compounds in DOM as key factors for soil quality in the Amazon basin [31].

In the context of the Amazon basin, the chemodiversity of DOM compounds is crucial for promoting soil organic matter decomposition and soil organisms’ activity [32]. Some DOM compounds can also serve as indicators of soil degradation [33,34]. The results of this study may suggest that in deforested sites, there is a significant decrease in microbial-derived compounds (fluorescence compounds **1**–**3**, biological index, and fluorescence index) and aliphatic compounds (including carbohydrates, lipids, and proteins/amino sugars). Conversely, there is an increase in the formation of aromatic compounds and/or a decrease in the decomposition of aromatic compounds. Our findings are consistent with previous studies that have described how land use reduces the accumulation of amino sugars and the decomposition of recalcitrant compounds [9,10,32]. The lower microbial carbon biomass and microbial respiration at deforested sites reduce oxygen supply and carbon substrate diffusion in the soil solution [1,35]. The impact of deforestation on soil physicochemical properties can also be influenced by changes in the chemodiversity of soil DOM compounds [36]. Microbial biomass and soil organic carbon tend to be concentrated in the primary Amazon rainforest. Thus, it is possible that microbial activities and the accumulation of microbial-derived compounds (fluorescence compounds **1**–**3**, biological index, and fluorescence index) per unit of soil mass are linked with plant diversity, root diversity, and the rhizobiome [37].

The abundance of polyphenolic and condensed aromatic compounds in the DOM showed the highest values in the deforested sites [38], suggesting that these aromatic compounds were released from soil aggregates as influenced by deforestation [39]. The practice of deforestation, aimed at converting forests into arable lands, encompasses various mechanical methods such as slash-cutting, soil scarification, mounding, and subsoiling. These practices induce multiple interactions among soil aggregates, exposing their inner compounds and consequently affecting soil dissolved organic matter (DOM) chemodiversity [1,39]. Intensive mechanical practices exacerbate soil disturbance by diminishing labile compounds and microbial activity [4]. Techniques like scarification and subsoiling induce significant changes in soil physical properties, thereby altering the state and function of tropical soils, including nutrient cycling, carbon stocks, and water retention [34]. Consequently, changes in DOM chemodiversity are expected, particularly in the abundance of polyphenolic and condensed aromatic compounds. Phenolic and polyphenolic compounds are crucial for wood degradation by microorganisms. However, mechanical practices during deforestation significantly impede microbial activity by reducing microbial respiration and biomass, as well as promoting the breakdown of aggregates, leading to the release of polyphenolic and condensed aromatic compounds. This contributes to the observed high abundance of these compounds in deforested sites.

Soils with high quality might have a lower formation of aromatic compounds, while degraded soils present a high abundance of these compounds [40]. In Acrisols, there is supposed to be a high sorption of phenolic and aromatic compounds due to their high mineral content. Thus, the relative abundance of aliphatic compounds and other labile compounds increases with mineral content [41]. The results of this study revealed differences among the land uses, especially deforestation, in the chemodiversity of soil DOM compounds. On the one hand, it could be considered that deforestation can significantly alter the chemodiversity of labile and microbial-derived compounds, consequently affecting soil quality and health [42]. On the other hand, the primary Amazon rainforest promotes the high abundance and diversity of such compounds, thereby enhancing soil physical–chemical properties and microbial activity [10]. We hypothesize that implementing soil organic matter management practices to enhance the abundance of labile compounds and microbial activity in deforested areas may mitigate or alleviate the adverse effects of deforestation on tropical soils, thereby facilitating reforestation efforts. Our results emphasize the importance of DOM chemodiversity knowledge in developing effective policies and incentives to mitigate the negative effects of deforestation.

Our hypothesis that plant diversity and root activity played key roles in regulating the chemodiversity of soil DOM in the Amazon basin was not supported for deforested sites. Overall, the chemodiversity of soil organic matter in the deforested sites was characterized by a significant decrease in aliphatic compounds, biological index, carbohydrates, fluorescence compounds **1**–**3**, fluorescence index, H/C ratio, lipids, NOSC, proteins/amino sugars, and SUVA_234_, as influenced by changes in the rhizobiome, litter deposition, and plant diversity [42]. The lack of active roots, litter, and plant cover (which protects soil aggregates from direct impacts of environmental factors) decreased the abundance of labile and microbial-derived compounds, soil aggregation (represented here by GMD and WAD), and soil organic carbon [10]. For the deforested sites, the lowest values of microbial carbon biomass and microbial respiration correspond to the lowest abundance of labile and microbial-derived compounds [43]. This finding is consistent with the work done by Yang et al. [44] and Zhou et al. [45], who reported similar trends in the chemodiversity of DOM influenced by environmental factors [46].

The process of deforestation leads to a significant reduction in the abundance of labile and microbial-derived compounds, resulting in a profound negative impact on microbial communities. This habitat simplification diminishes both habitat provision, such as soil aggregates, and nutrient supply, consequently lowering microbial carbon biomass and microbial respiration rates. Numerous studies have documented the detrimental effects of land use changes on soil microorganism activity, which is often influenced by plant diversity and soil organic matter content [4,9]. However, our study offers a deeper insight into the primary factor behind this decline in microbial activity: the decreased abundance of labile and microbial-derived compounds.

In tropical soils, a high number of key processes contribute to soil organic matter accumulation and the chemodiversity of compounds in the soil solution, including mineral sorption, microbial senescence, soil organic matter dissolution, and microbial and litter production [47]. Despite the wide variation in DOM and the complex relationship between DOM compounds and deforestation, our results have shown that habitat simplification can account for most of the chemical composition in the DOM, implying that the chemodiversity of deforested sites can reduce microbial activity, nutrient cycling, and soil quality [48]. These results are consistent with findings from Wu et al. [49] and Castañeda-Gómez et al. [43], who reported a low abundance of labile compounds in disturbed soils. In disturbed soils, these authors reported a high decline in microbial-derived compounds, which in turn decreases microbial activity and soil quality.

## 5. Conclusions

Deforestation showed the highest negative impacts on the chemodiversity of DOM compounds, soil physical–chemical properties, and microbial activity in Acrisols under field conditions in the Amazon basin. Our findings suggest that deforestation decreases aliphatic compounds, biological index, carbohydrates, fluorescence compounds **1**–**3**, fluorescence index, H/C ratio, lipids, NOSC, proteins/amino sugars, and SUVA_234_, while it increases the abundance of polyphenolic and condensed aromatic compounds. Additionally, we observed losses in soil physical–chemical properties and microbial activity as influenced by deforestation. The results of this study highlight the importance of considering the primary Amazon rainforest and the negative impacts of deforested sites on soil quality and soil chemodiversity.

## Figures and Tables

**Table 1:** The site locations, geographic coordinates, and elevations for the studied sites in Brazil’s Legal Amazon.

Studied Site	Location	Geographic Coordinates	Elevation (m.a.s.l)
Site 1	Cruzeiro do Sul, Acre	7°37′17″ S 72°42′43″ W	192
Site 2	Rio Branco, Acre	9°57′11″ S 67°52′18″ W	165
Site 3	Boca do Acre, Amazonas	8°45′12″ S 67°23′09″ W	104
Site 4	São Sebastião do Uatumã, Amazonas	2°40′47″ S 58°02′49″ W	69
Site 5	Manaus, Amazonas	2°58′57″ S 59°55′53″ W	86
Site 6	Manicoré, Amazonas	5°47′00″ S 61°15′37″ W	64
Site 7	Cerejeiras, Rondônia	13°10′07″ S 61°14′29″ W	194
Site 8	Porto Velho, Rondônia	8°22′33″ S 63°30′16″ W	70
Site 9	Boa Vista, Roraima	2°49′32″ N 60°38′05″ W	63
Site 10	Caracaraí, Roraima	0°45′35″ N 60°57′05″ W	60

**Table 2:** The chemodiversity of soil dissolved organic matter (DOM) in soil samples (mean ± sd) across Brazil’s Legal Amazon as influenced by land use.

Variables	Primary Amazon Rainforest	Pasture	Deforested Site
Aliphatic compounds (%)	20.6 ± 1.3 a	15.2 ± 0.3 b	2.1 ± 0.4 c
Biological index	4.6 ± 0.3 a	1.2 ± 0.4 b	0.2 ± 0.1 c
Carbohydrates (%)	28.2 ± 2.1 a	15.2 ± 1.3 b	2.1 ± 0.9 c
Condensed aromatic compounds (%)	25.2 ± 2.5 b	30.8 ± 2.8 a	31.1 ± 3.7 a
Fluorescence compounds **1**–**3**	4.2 ± 0.5 a	2.9 ± 0.4 b	1.5 ± 0.3 c
Fluorescence compounds **4**	−4.1 ± 0.2 b	−4.2 ± 0.3 b	−2.1 ± 0.2 a
Fluorescence index	3.1 ± 0.4 a	1.9 ± 0.2 b	0.5 ± 0.1 c
H/C ratio (%)	2.4 ± 0.2 a	1.2 ± 0.1 b	0.4 ± 0.2 c
Highly unsaturated and phenolic compounds (%)	23.4 ± 3.4 b	24.8 ± 2.7 b	60.8 ± 5.4 a
Lipids (%)	19.3 ± 3.2 a	16.4 ± 1.7 b	9.3 ± 3.1 c
Nominal oxidation stage of carbon (%)	2.5 ± 0.4 a	1.3 ± 0.5 b	0.2 ± 0.1 c
O/C ratio (%)	1.2 ± 0.3 a	1.1 ± 0.2 a	1.2 ± 0.3 a
Polyphenolic compounds (%)	24.3 ± 3.7 b	25.8 ± 2.1 b	30.7 ± 1.7 a
Proteins/amino sugars (%)	41.3 ± 5.3 a	19.1 ± 3.1 b	0.9 ± 0.2 c
Specific ultraviolet absorbance at 254 nm	3.7 ± 1.1 a	2.1 ± 0.3 b	1.9 ± 0.4 b

Four fluorescence components were identified, including three humic-like components (components 1–3) and one protein-like component (component 4). Within land uses, the same letters represent no significant differences by Bonferroni’s test (*p* < 0.05).

**Table 3:** Soil physical–chemical properties of soil samples (mean ± sd) across Brazil’s Legal Amazon as influenced by land use.

Soil Properties	Primary Amazon Rainforest	Pasture	Deforested Site
Bulk density (g cm^−3^)	0.92 ± 0.05 c	1.11 ± 0.16 b	1.27 ± 0.41 a
Geometric mean diameter (mm)	2.57 ± 0.34 a	2.38 ± 0.27 a	1.13 ± 0.17 b
Weighted average diameter (mm)	3.04 ± 0.31 a	2.96 ± 0.41 a	1.86 ± 0.19 b
Sand (g kg^−1^)	238.39 ± 21.23 a	241.67 ± 19.17 a	240.19 ± 12.26 a
Silt (g kg^−1^)	524.70 ± 17.01 a	520.91 ± 19.16 a	529.13 ± 21.19 a
Clay (g kg^−1^)	236.91 ± 21.58 a	237.42 ± 19.46 a	230.68 ± 22.93 a
Soil pH	3.74 ± 0.28 c	5.18 ± 0.31 b	6.23 ± 0.18 a
SOC (g kg^−1^)	43.12 ± 3.45 a	27.93 ± 4.91 b	6.45 ± 1.29 c
Microbial C biomass (g C kg^−1^)	543.87 ± 2.98 a	72.38 ± 0.82 b	14.98 ± 1.02 c
Microbial respiration (mg kg^−1^ h^−1^)	0.065 ± 0.002 b	0.034 ± 0.004 a	0.008 ± 0.001 c

Within land uses, the same letters represent no significant differences by Bonferroni’s test (*p* < 0.05).

## Data Availability

The raw data supporting the conclusions of this article will be made available by the authors on request.
